# Umbilical Cord Diseases Affecting Obstetric and Perinatal Outcomes

**DOI:** 10.3390/healthcare11192634

**Published:** 2023-09-27

**Authors:** Gabriele Tonni, Mario Lituania, Alessandro Cecchi, Elisa Carboni, Serena Resta, Maria Paola Bonasoni, Rodrigo Ruano

**Affiliations:** 1 Department of Obstetrics and Gynecology, Istituto di Ricovero e Cura a Carattere Scientifico (IRCCS), AUSL Reggio Emilia, 42100 Reggio Emilia, Italy; 2 Preconceptional and Prenatal Pathophysiology, Department of Obstetrics and Gynecology, E.O. Ospedali Galliera, 16128 Genoa, Italy; mariolituania48@gmail.com; 3 Department of Obstetrics and Gynecology, Regional Prenatal Diagnostic 2 Level Center, ASUR Loreto Hospital, 60025 Loreto, Italy; cecchi.alessandro@gmail.com (A.C.); elisacarboni@hotmail.com (E.C.); 4 Department of Obstetrics and Gynecology, Fondazione Policlinico Tor Vergata, University of Tor Vegata, 00133 Rome, Italy; serena.resta@aslroma1.it; 5 Department of Pathology, Santa Maria Nuova Hospital, Istituto di Ricovero e Cura a Carattere Scientifico (IRCCS), AUSL Reggio Emilia, 42100 Reggio Emilia, Italy; paolabonasoni@yahoo.it; 6 Division of Maternal-Fetal Medicine, Department of Obstetrics, Gynecology & Reproductive Sciences, Miller School of Medicine, University of Miami, Miami, FL 33136, USA; rodrigoruano@hotmail.com

**Keywords:** umbilical cord anomalies, maternal–fetal complications, fetal mortality, neonatal mortality, perinatal mortality, adverse perinatal outcomes

## Abstract

Background: (1) The aim of this article is to describe the physiopathology underlying umbilical cord diseases and their relationship with obstetric and perinatal outcomes. (2) Methods: Multicenter case series of umbilical cord diseases with illustrations from contributing institutions are presented. (3) Results: Clinical presentations of prenatal ultrasound findings, clinical prenatal features and postnatal outcomes are described. (4) Conclusions: Analysis of our series presents and discusses how umbilical cord diseases are associated with a wide variety of obstetric complications leading to a higher risk of poor perinatal outcomes in pregnancies. Knowing the physiopathology, prenatal clinical presentations and outcomes related to umbilical diseases allow for better prenatal counseling and management to potentially avoid severe obstetric and perinatal complications.

## 1. Introduction

The correct function of the placenta is paramount in providing adequate blood flow to the fetus through the umbilical cord, favoring progressive growth and overall survival. The umbilical cord is formed by three vessels of different sizes: the larger vessel is the umbilical vein (supplying arterial blood) surrounded by two smaller arteries (supplying venous blood) that are characterized by a spiral course along the umbilical vein. These vessels are surrounded by an extracellular matrix (ECM) called Wharton’s jelly, the function of which is to protect the umbilical vessels. The umbilical cord provides the necessary oxygenated blood flow to the fetus. Different diseases may cause alterations in the structure of the cord leading to abnormalities such as stricture, hypo/hypercoiling, or vessel rupture that will generate a perturbation in the gas exchange to the fetus that may jeopardize fetal well-being [1], and cord anomalies can induce stillbirth in 3.4% to 20% of cases [2]. In 2022, the global stillbirth rate was 13.9 stillborn babies per 1000 total births [3]. Many factors contribute to stillbirth, but accurate placental and umbilical cord examination can reveal the cause of death in 11–65% of cases [4]. Adverse fetal or perinatal outcomes may occur in the cases of nuchal cord, cord entanglement, true knot, abnormal coiling and/or length, and cord prolapse [5]. Understanding the underlying causes of umbilical cord pathologies may be helpful in identifying the etiopathogenesis and subsequent treatment of adverse perinatal outcomes [6,7]. Maternal diseases such as preeclampsia (PE) may be associated with fetal growth restriction (FGR), in turn resulting in a higher risk of adverse obstetric and perinatal outcomes such as stillbirths or long-term morbidity [8,9,10]. A recent proteomic investigation demonstrated that in fetuses with FGR or PE with superimposed FGR, the most significant changes occurring to the umbilical cord involved proteins associated with Wharton’s jelly and inflammatory and angiovascular processes [11].

In this review, we focused on describing the main umbilical cord diseases, from the etiopathogenesis to the prenatal two-dimensional (2D) or three-dimensional (3D) ultrasound diagnosis, including the clinical and histological evaluation and outcome.

## 2. Materials and Methods

This paper describes the clinical presentations of a multicenter case series of umbilical cord diseases that can affect pregnancies. The clinical presentations and illustrations are reported from case series from different obstetric tertiary care centers. The prenatal ultrasound diagnosis and clinical features as well as the obstetric and perinatal outcomes are described. Since the data presented were derived from ultrasound examinations following the national guidelines for ultrasound screening during pregnancy, there is no need for an Institutional Review Board approval at a single center. Electronic search based on keywords has been investigated and conducted on PubMed/Medilne and SCOPUS from inception to 21 September 2023.

## 3. Umbilical Cord Diseases

### 3.1. Cord Coiling 

**Figure 1:**
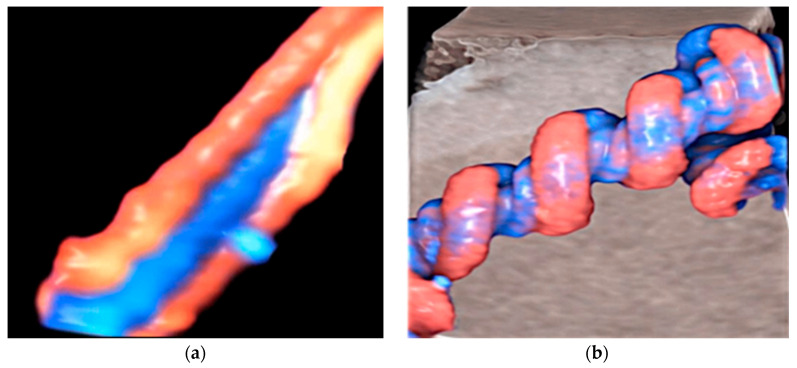
(**a**) Two-dimensional ultrasound with HD-Flow color Doppler showing a hypocoiled umbilical cord and (**b**) a hypercoiled umbilical cord.

The umbilical cord (UC) is the vital connection between the fetus and the placenta, carrying oxygenated blood through the umbilical vein and removing deoxygenated blood via the umbilical arteries. The characteristic helical coiling of the cord helps to prevent twisting, compression, and traction, and it is also involved in regulating the blood flow to and from the placenta. The umbilical coiling index (UCI) specifies the number of coils, which normally is one coil every five centimeters [12,13]. A hypocoiled cord is defined as one coil per 10-cm segment, whereas hypercoiling presents more than three coils per 10 cm. Both conditions may be associated with adverse fetal outcomes, including FGR and intrauterine fetal death (IUFD) [14,15]. The umbilical coil index (UCI) is defined as the total number of vascular coils divided by the cord’s length [16,17]. Hypocoiled cords are defined as UCI < 10th percentile (0.26 coils/cm), while hypercoiled cords are defined as UCI > 90th percentile (0.46 coils/cm) [18]. A low UCI has been significantly associated with stillbirth, preterm labor, and oligohydramnios, while a high UCI has been associated with intrauterine fetal growth restriction (FGR) [19]. Both hypocoiled and hypercoiled cords have been associated with fetal heart rate abnormalities during labor and low birth weight [16,17]. A severe hypercoiled cord can result in IUFD if the umbilical ring is constricted [15] (Figure 1a,b).

**Figure 2:**
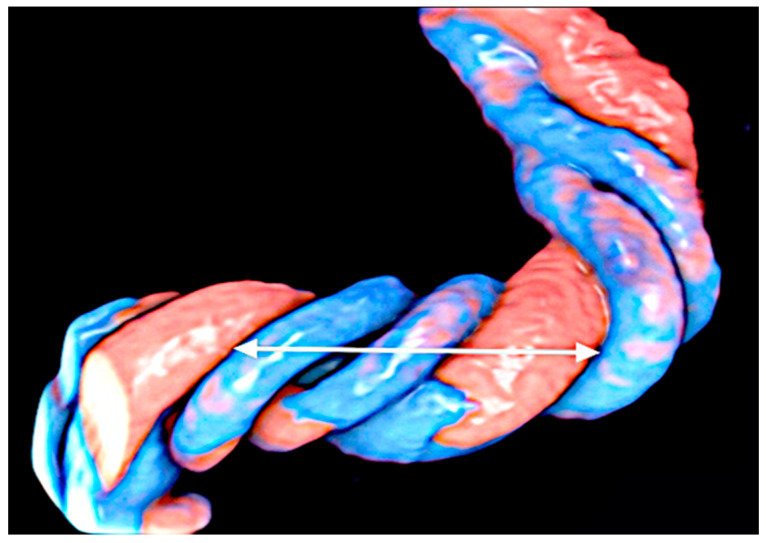
Umbilical cord displayed in HDlive Flow. Measurement of sonographic umbilical cord index (UCI): 1/one coil. One coil corresponds to a complete coil of 360° of the umbilical vessels around each other measured in centimeters. Basically, the distance between the inner edge of the umbilical artery wall to the outer edge of the arterial wall itself should be measured up to the next spiral (left–right arrow). Interestingly, in twin–twin transfusion syndrome, the recipient usually displays a hypercoiled cord, probably having been developed as a protective mechanism against volume overload [24] (Figure 2 and Figure 3).

Hypercoiling is frequently associated with a long cord, and as an expression of compromised fetal blood flow, fetal vascular malperfusion (FVM) is usually seen during placental examination. These histological features include thrombosis in fetal vessels, avascular villi, villous-stromal karyorrhexis, and normoblastemia [14,20]. Massive fibrin deposition has also been observed [21]. Though a hypercoiled cord has been reported as a recurrent finding, no definitive genetic cause has been established [20,21,22]. Although the etiology of abnormal cord coiling is still unknown, hemodynamic changes and fetal movements may be a cause. Short cords and fewer coils are found in conditions associated with reduced fetal movements such as skeletal dysplasia or muscular disorders, as well as intrauterine physiological space limitation (i.e., oligohydramnios, twin pregnancy, prolonged rupture of membranes) [20]. In long and hypercoiled cords, Poiseuille’s equation is applied, considering that blood flow speed is inversely proportional to the vascular length [23]. Therefore, excessive blood flow can possibly be regulated over time by lengthening the hypercoiling of the cord as an adaptive condition. Interestingly, in twin–twin transfusion syndrome, the recipient usually displays a hypercoiled cord, probably having been developed as a protective mechanism against volume overload [24] (Figure 2).

**Figure 4:**
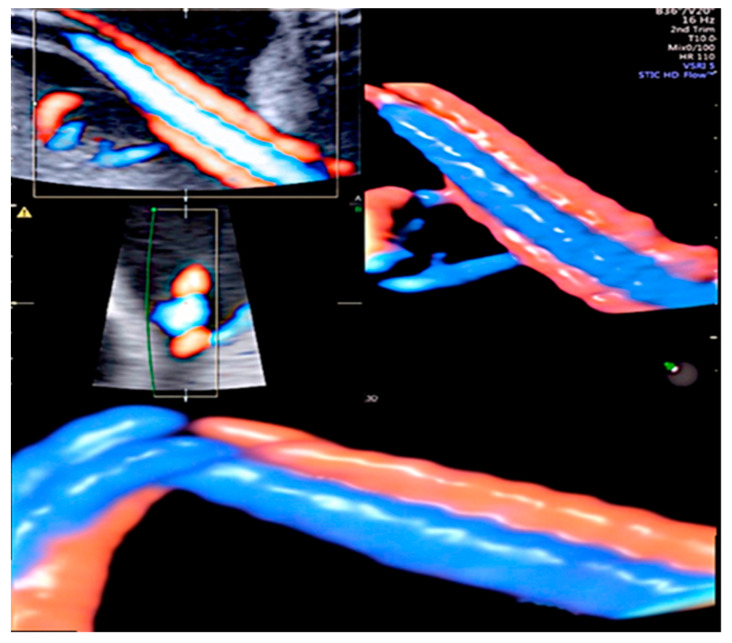
Umbilical cords displayed in HDlive Flow. Uncoiled cord: two straight umbilical arteries with UCI equal to 0.

**Figure 5:**
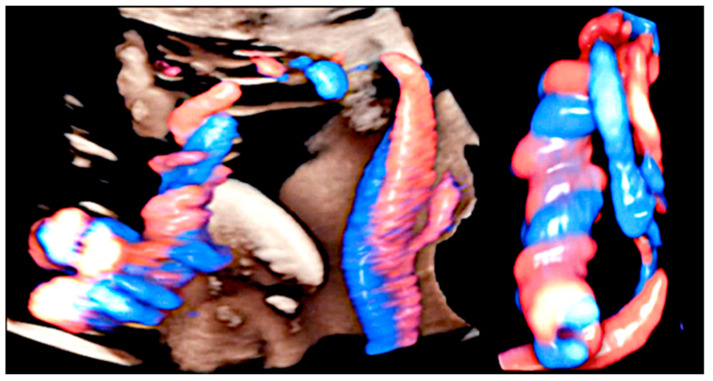
Three-dimensional ultrasound with HDlive Flow can detect hypercoiled and uncoiled cord segments in the same fetus.

In extreme cases, the umbilical cord can be “uncoiled” and severely reduced or absent coiling may be localized, causing a reduction in the diameter of the cord, which has a constricting and/or twisting effect, leading to fetal vascular malperfusion [23] (Figure 4 and Figure 5).

### 3.2. True Umbilical Cord Knot 

**Figure 6:**
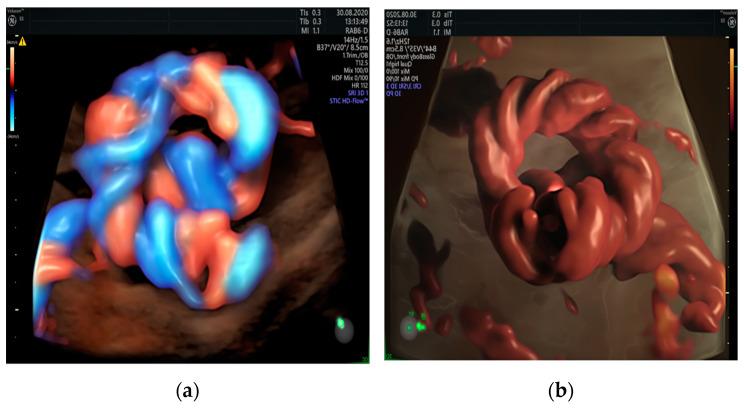
True umbilical cord knot diagnosed at 21 weeks of gestation using spatiotemporal image correlation (STIC) using HD-Flow (**a**) and “glass-body” mode in iFlow (**b**).

**Figure 7:**
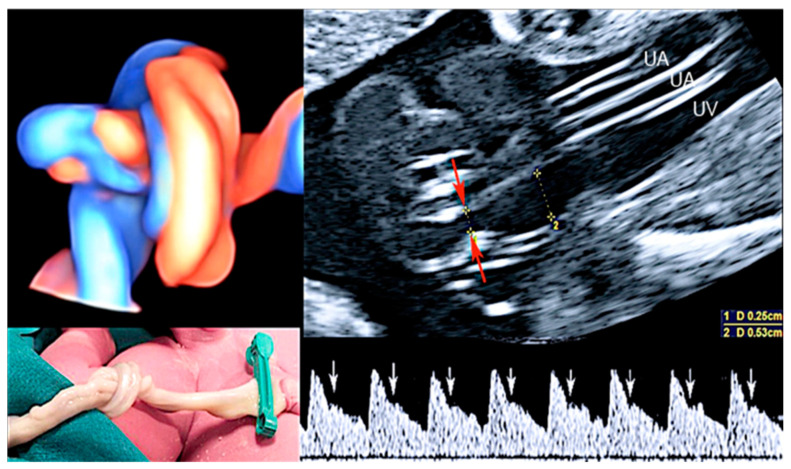
Umbilical cord knot associated with notching (white arrows) in the umbilical artery (UA) Doppler waveform. The plausible cause for notching in the UA is constriction: UA diameter section: 2.5 mm (red arrows), UV diameter section: 5.3 mm (yellow calipers). Waveform notching is determined by flow separation induced by constriction. The notching is not present in cases of less than 75% constriction and disappears as the vortex wave is attenuated at distances downstream of the constriction. Notching in the UA Doppler waveform should be considered important even in the absence of increased peak systolic velocity of the fetal mean cerebral artery (MCA) because it might indicate impending intrauterine fetal death (IUFD).

A true knot of the umbilical cord is a rare obstetric condition with an incidence of roughly 1.2% of all pregnancies. It significantly increases the rate of stillbirth, with an odds ratio (OR) of 3.96 [18], and it can be associated with adverse perinatal outcomes such as IUGR, preterm birth, low Apgar scores, and stillbirth [25,26]. True knots have been reported to lead to a four-fold increase in IUFD due to vascular compression as long as the knot tightens [27]. They are more common in mothers with an advanced age, multiparity, previous miscarriages, polyhydramnios, and diabetes [18,26]. Co-occurrence of nuchal chord can be a contributory negative prognostic factor [28]. True knots tend to be formed in early pregnancy when the amniotic fluid is abundant and the fetus can move easily. They are more frequently detected in long cords and in male fetuses [29]. Other adverse outcomes related to true knot are SGA, preterm birth, an Apgar score < 7 at the 5th minute, and NICU admission [18]. The visualization of a segment of the umbilical cord closely surrounded by another loop of cord, known as a “hanging noose sign”, is a highly specific finding of a tight knot, while the true knot itself can be easily detected by 3D ultrasound [30] (Figure 6a,b and Figure 7).

### 3.3. Umbilical Cord Stricture 

**Figure 8:**
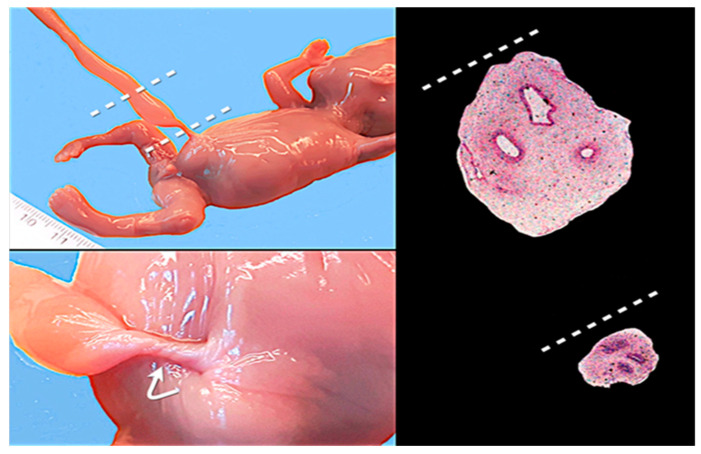
A reduced or absent coiling may be localized, causing a reduction in the diameter of the cord, with a constricting and/or twisting effect (indicated by the curved arrow). This phenomenon is more frequent near the umbilical ring. The reduced or absent coiling may be associated with a reduction in Wharton’s jelly.

Umbilical cord stricture is an unusual cause of IUFD. The exact etiology is still unknown, but as the stricture is focally characterized by reduced Wharton’s jelly and the fetal movements induce cord twisting, cord vessels easily undergo stretching and the blood flow is blocked, with consequent fetal demise [31]. This possible explanation goes along with the theory that during the second trimester the fetus is particularly active, favoring localized cord constriction. On the other hand, localized paucity of Wharton’s jelly determines cord weakness and the fetus can easily rotate around this destabilized point. The site of cord torsion is typically at the fetal end and induces vascular restriction. The progressive reduction in fetal blood flow determines firstly hypoxia, acidosis, and then death [32] (Figure 8).

**Figure 9:**
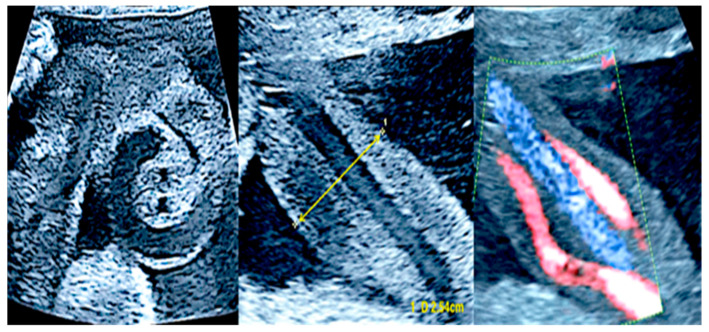
Two-dimensional ultrasound and color Doppler. Umbilical cord diameter anomaly: a thick cord depends on the amount of Wharton’s jelly that has formed and deposited in the cord (diameter section (yellow calipers): 25.4 mm). It may be associated with maternal diabetes, fetal hydrops, polyhydramnios, and coiling anomalies (uncoiled/hypocoiled).

**Figure 10:**
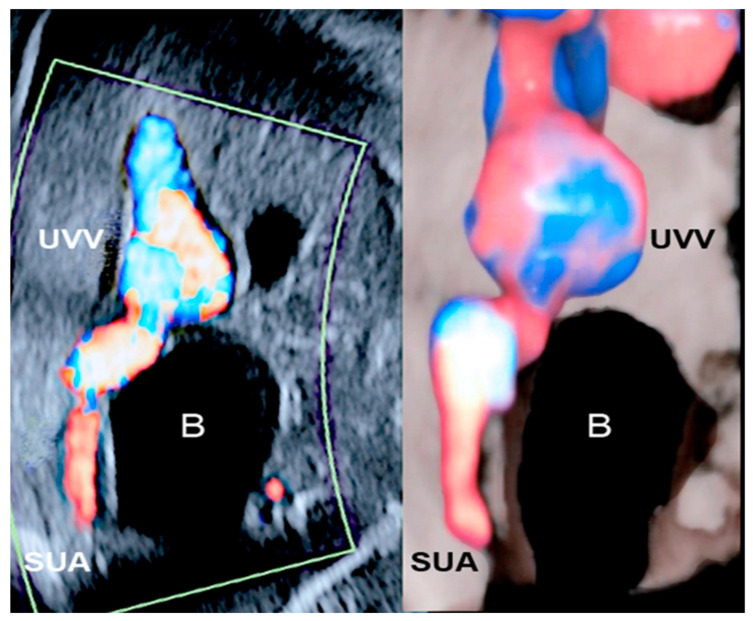
Color Doppler and 3D ultrasound with “glass-body” mode. Absent left umbilical artery. Single umbilical artery (SUA) associated with intra-abdominal umbilical vein varix (UVV) and absence of the ductus venosus. The umbilical vein connects to the inferior vena cava in the suprahepatic area. In the presence of a large varix and bidirectional turbulence flow, the risk of developing a thrombus is higher. (Abbreviation. B: bladder).

Opposite to what is seen in the case of umbilical cord stricture is a thick cord due to a higher concentration of Wharton’s jelly. Considering that a variation in the amount of Wharton’s jelly in the umbilical cord may be congenital, a thick umbilical cord may also be seen early in gestation, and be associated with maternal diabetes, fetal hydrops, polyhydramnios, and coiling anomalies [33] (Figure 9 and Figure 10).

### 3.4. Single Umbilical Artery 

Single umbilical artery (SUA) is detected in around 0.5% of second trimester morphology screenings, and the left umbilical artery is the one most commonly missing [34]. SUA is typically concomitant with fetal anomalies with the following occurrence: cardiovascular, urogenital, musculoskeletal, and cerebral. In these cases, aneuploidy is frequent [35]. The pathogenesis of SUA involves different proposed mechanisms that may contribute and interact at different times. For example, it may derive from an early vascular accident or a secondary atrophy. In fact, the development of definitive umbilical arteries happens very early in embryogenesis, as demonstrated by the close relationship between SUA and body stalk defects. In the phase of blastogenesis, within the first four weeks after conception, failure or delay of cord formation may explain the occurrence of common anomalies such as esophageal atresia, imperforate anus, and renal and vertebral defects [36,37]. When SUA is seen as an isolated finding during the ultrasound examination, the antenatal and neonatal outcome is usually favorable; if it is detected as associated with other abnormal structural defects, the fetus has a higher risk of chromosomal abnormalities and adverse outcomes during the neonatal period [30,38,39] (Figure 10).

### 3.5. Supernumerary Vessels 

**Figure 11:**
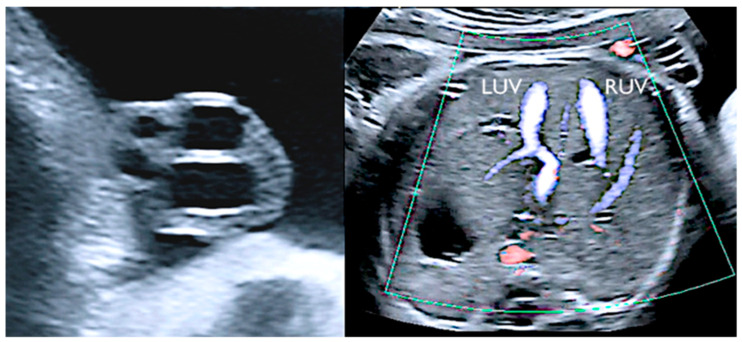
Two-dimensional ultrasound and color Doppler reveals the presence of four vessels in the umbilical cord. Axial plane of the fetal abdomen highlights the left (LUV) and right (RUV) umbilical veins.

The term “supernumerary vessels” designates more than three vessels in the umbilical cord. The additional vessel may be one artery or one vein. In the case of a supernumerary vein, this vessel derives from the persistence of the right umbilical vein, accompanied by a patent left one. The early venous system appears around the fourth gestational week and is composed of three symmetric paired veins: umbilical, vitelline, and cardinal. Before the sixth week of gestation, the whole right umbilical vein and the proximal segment of the left umbilical vein involute, while there is persistence of the distal part of the left umbilical vein. Failure in regression of the right umbilical vein gives rise to the extra venous vessel [37]. A supernumerary vein has been found to be associated with cleft lip and palate, cardiovascular and gastrointestinal malformations, and fetal hydrops [40]. Embryological development of the umbilical arteries may enlighten the underlying mechanism of the finding of an additional artery in the cord, namely three arteries and one vein. During the third week of gestation, the umbilical arteries emerge as ventral divisions of the paired dorsal aortas. After their fusion, the umbilical arteries fuse with the descending aorta, remaining as two lateral branches in its distal part. Simultaneously, the allantois is surrounded by an arterial plexus that merges in a single artery, lengthening throughout the body stalk. This allantoic artery progressively atrophies as the right and left umbilical arteries grow along the body stalk, and it ultimately unites with the right and left umbilical artery to constitute the interarterial anastomosis at the base of cord insertion. Throughout this process, an additional umbilical artery may result from the failure of fusion of the primitive umbilical arteries with the descending aorta or a deficiency in the coalescence of the arterial plexus to form the allantoic artery. This vessel may also fail to merge with the right and left umbilical artery. Another possibility is the splitting of an umbilical artery during its embryological development [37]. Three arteries and one vein are a highly uncommon combination, and thus the incidence of associated anomalies is difficult to ascertain. It has been described in a stillbirth with no further malformations [41] (Figure 11).

### 3.6. Umbilical Cord Cysts

**Figure 12:**
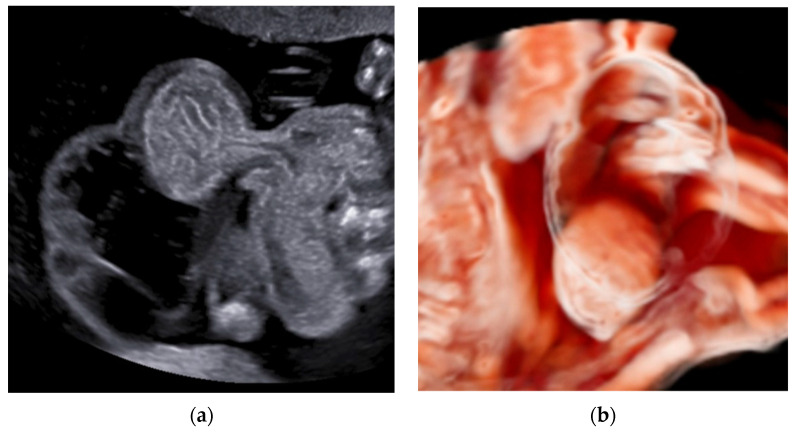
Two-dimensional ultrasound (**a**) and 3D ultrasound using Crystal Vue showing a fetus with exomphalos associated with a large umbilical cord cyst detected at 16 weeks’ gestation (**b**).

Umbilical cord cysts are classified as true cysts or pseudocysts. True cysts can originate from the remnants of the allantois or the omphalomesenteric duct. They are epithelial-lined and are found close to the fetal cord insertion. Within true cysts, amniotic inclusion cysts may coexist [42,43]. Urachal cysts tend to disappear by the end of the third trimester, but exomphalos, persistent urachus, and uropathy may be associated [44]. Large cysts may affect the fetus by cord compression, reducing blood flow and favoring thrombosis [42]. Pseudocysts are typically situated near the fetal cord insertion. They derive from the focal degeneration of Wharton’s jelly and lack epithelial tissue [42,43]. Umbilical cord cysts have been linked to aneuploidies, especially trisomy 13 and 18, and malformations, such as exomphalos, in different series [45] (Figure 12).

### 3.7. Angiomyxoma/Hemangioma of the Umbilical Cord 

**Figure 13:**
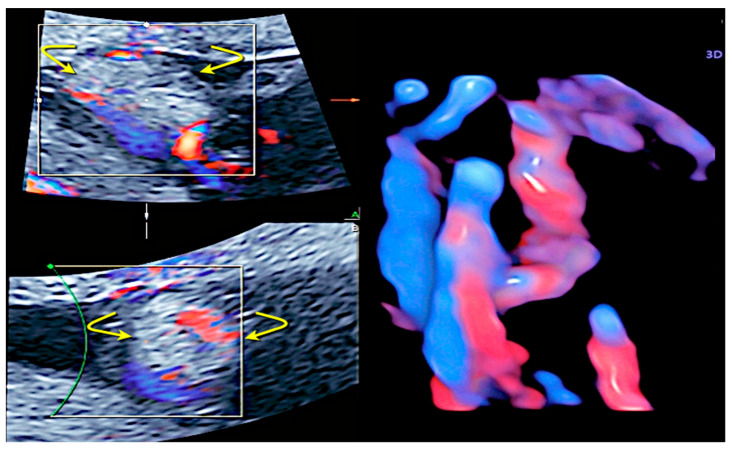
Umbilical cord angiomyxoma detected at 16 weeks’ gestation. Angiomyxoma is located at the level of the cord insertion into the placenta. Multiplanar display color Doppler and 3D ultrasound with “glass-body” rendering mode shows the angiomyxoma and its effects on the cord vessels. It appears as a rounded echogenic area (indicated by yellow curved arrows) which envelops, squeezes, and sometimes stretches the umbilical vessels.

Angiomyxoma is a benign vascular neoplasm of the umbilical cord. It is composed of a capillary proliferation enclosed in a fibromyxoid stroma, which is intrinsic to the cord and not produced by the lesion. Degeneration of Wharton’s jelly may occur, resulting in pseudocystic changes [46]. Associated congenital vascular anomalies have been described in association with angiomyxoma, but no causative link has been defined to date. Infantile hemangiomas have been reported, especially of the skin, liver, intestine, abdominal wall, and vulva [47]. Elevated maternal serum alpha-protein (MS-AFP) and human chorionic gonadotropin (MS-hCG) levels have been reported and in such cases an extended prenatal ultrasound should exclude possible imaging referable to hemangiomas [48,49]. Furthermore, an adverse perinatal outcome is associated with high perinatal mortality and morbidity, with a fetal mortality rate ranging in different case series from 35% to 54% [50,51]. Angiomyxoma may be a risk factor for FGR and IUFD due to impaired umbilical cord circulation. Polyhydramnios and non-immune fetal hydrops may also occur [52]. In congenital hemangioma, syndromic and non-syndromic capillary malformation mutations have been found on the guanine nucleotide-binding protein subunit alpha-11 (GNA11) or guanine nucleotide-binding protein G(q) subunit alpha (Gαq) (GNAQ) genes [53]. Moreover, in non-syndromic superficial angiomyxoma, the absent or reduced expression of cAMP-dependent protein kinase type I-alpha regulatory subunit (PRKAR1A) has been observed [54]. These genetic correlations should be further analyzed in the case of umbilical cord angiomyxoma (Figure 13).

## 4. Conclusions

Following analysis of the medical literature and that of our case series, a number of considerations can be drawn. (1) It is important to understand the physiopathology, clinical presentations, and outcomes of different umbilical cord pathologies in order to correctly counsel the parents-to-be during antenatal care. (2) Two-dimensional and 3D ultrasound may enhance the quality of prenatal imaging, improving the prenatal ultrasound diagnosis. (3) An enhanced prenatal sonographic diagnosis is clinically important to prevent or avoid obstetric and perinatal complications and assist physicians in planning and delivering the best antenatal management in the case of umbilical cord diseases. (4) Where necessary, in the case of stillbirths or other obstetric and perinatal adverse outcomes, a fetal pathology is mandatory and may lead to better recognition and understanding of the underlying cord pathologies and potentially prevent medico-legal issues [55]. 

## Figures and Tables

**Figure 3:**
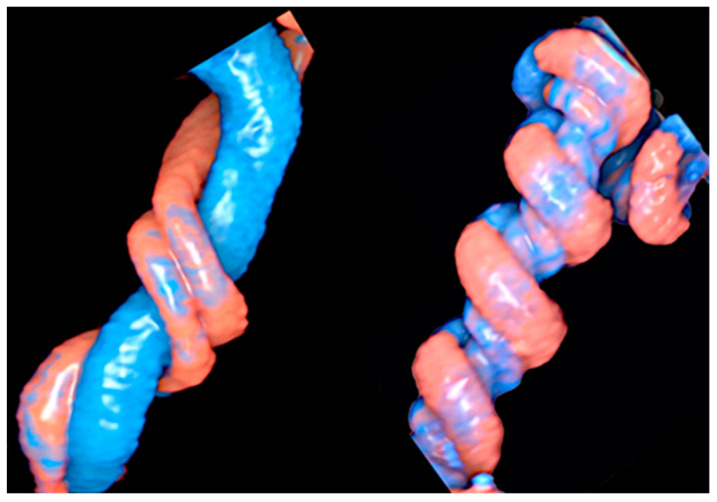
Umbilical cords displayed in HDlive Flow. The cord is hypocoiled if UCI is lower than the 10th centile for gestational age (on the left); hypercoiled if UCI is above the 90th centile for gestational age (on the right).

## Data Availability

There are no data associated with this publication.
